# ﻿Extracting specimen label data rapidly with a smartphone—a great help for simple digitization in taxonomy and collection management

**DOI:** 10.3897/zookeys.1233.140726

**Published:** 2025-03-26

**Authors:** Dirk Ahrens, Alexander Haas, Thaynara L. Pacheco, Peter Grobe

**Affiliations:** 1 Museum A. Koenig, Leibniz Institute for the Analysis of Biodiversity Change (LIB), Adenauerallee 127, 53113 Bonn, Germany Leibniz Institute for the Analysis of Biodiversity Change Bonn Germany; 2 Museum of Nature Hamburg, Leibniz Institute for the Analysis of Biodiversity Change, Martin-Luther-King-Platz 3, 20146 Hamburg, Germany Leibniz Institute for the Analysis of Biodiversity Change Hamburg Germany

**Keywords:** Artificial intelligence, citizen science, collection digitization, data science, label transcription, labels, taxonomic impediment, taxonomic revisions

## Abstract

We provide short tutorials in how to read out specimen label data from type- as well as handwritten labels in a rapid and easy way with a mobile phone. We apply them in general, but test them in particular for insect specimen labels, which are generally quite small. We provide alterative procedure instructions for Android and Apple based environments, as well as protocols for single and bulk scans. We expect that this way of data capture will be of great help for a simple digitization in taxonomy and collection management, independent from large industrial digitization pipelines. By omitting the step of taking/maintaining images of the labels, this approach is more rapid, cheaper, and environmentally more sustainable because no storage with carbon footprint is required for label images. We see the biggest advantage of this protocol in the use of readily available commercial devices, which are easy to handle, as they are used on a daily basis and can be replaced at relatively low cost when they come into (informatic) age, which is also a matter of cyber security.

## ﻿Introduction

Currently, there are immense efforts underway to digitize natural history collections on a large scale, including the associated information and metadata (e.g., Smith and Blagoderov 2012; Hardisty et al. 2020a, b; Belot et al. 2023; Groom et al. 2023; Ong et al. 2023). In these endeavors, among other things, the automatic capture of label data plays a central role (e.g., Beaman et al. 2006; Heidorn and Wei 2008; Lafferty and Landrum 2009; Granzow-de la Cerda and Beach 2010; Haston et al. 2012; Agarwal et al. 2018; Alzuru et al. 2019, 2020; Alzuru 2020; Owen et al. 2020; Belot et al. 2023; Zhang 2023; Takano et al. 2024). However, many of these very promising activities have been for a long time exclusive to large companies, museums or institutions with specialized technical infrastructure, and specially trained staff (e.g., Blagoderov et al. 2012) for the highly customized implementations used (e.g., https://picturae.com/).

Most of the current digitization initiatives aim at a one-go retro-digitization of large collections (Engledow et al. 2018; Hardisty et al. 2020a, b; Helminger et al. 2020; De Smedt et al. 2024). However, this approach comes with limitations: 1) collections are continuously growing and developing (see also Balke et al. 2013); and 2) the scientific community produces a large amount of high-quality biodiversity data independently of the collection institutions with their ongoing research on the specimens, in which amateur scientists are also largely involved (Löbl et al. 2023). The latter is achieved by the often-remote study of the collection material, far away from collections and large digitization pipelines. Especially in insects, taxonomic specialists are rare, and specimens are often loaned by shipment overseas to obtain best identifications from world-leading specialists. In this, working processes are quite different from those of vertebrates or plants in often leading in new methodologies, such as large-scale digitization. However, these data often do not end up in big data repositories, also due to the lack of time and stimulus, as well as the work-overload of taxonomists.

Therefore, more flexible solutions are needed that allow for more efficient data processing and speed up biodiversity/species discovery and help to overcome taxonomic impediment. This would be perfectly in line with the idea of integrating specimen databases and revisionary systematics (Schuh 2012). Advantages of a revision-based digitization (see also Meier and Dikow 2004), i.e., that biodiversity data come from taxonomic revisionary studies rather than from uncritical retro-digitizing of museum specimen data, are the following (based on Meier and Dikow (2004) and Schuh (2012)): 1) the data are provided in association with the most accurate identifications, 2) the data have the most complete taxonomic and geographic coverage, 3) the data satisfy these points in a cost-effective way, and 4) the occurrence data and images are citable and acknowledgeable. This last point enables errors to be retracted and corrected).

Recently, we found that mobile devices, which nowadays are used by almost everyone, can be of assistance in speeding up data collection and digitization, including biodiversity discovery. By simple experimentation, we discovered that mobile phones can be used in association with cloud-like environments (such as Google Workspace or Apple iCloud). Because we think that these workflows will be useful to a larger audience, we prepared this short paper on how to rapidly and easily read out specimen label data using a smartphone.

Most digitization approaches capture digital metadata (e.g., labels) with the intermediate step of digital images (Nelson et al. 2012). This comes with difficulties and the considerable cost of image processing and storage (Tann and Flemons 2008; Hardisty et al. 2020b). It is more sustainable to skip this step, with the greatest benefit to cost, if data can be read and spell-checked at the same moment without the burden of intermediate images, which are scientifically and practically unnecessary for non-type specimens.

## ﻿Material and methods

### ﻿Resources needed

1) A mobile or smartphone; a recent model with macrophotography capacity.

2) A stable internet connection.

3) A computer connected with internet and logged into a Google account (via Google Chrome Browser) or AppleID account.

4) A database or text file to insert the specimen data.

5) "*Google Lens*" or "*Google Translate*" to be installed on the mobile phone.

For our testing here, we used a Motorola Edge 30 Neo (system: Android ver. 14), a Motorola G5g Plus (system: Android ver. 10/11), a Motorola G82 G5g (system: Android ver. 13), and an iPhone 15 Pro Max (system: iOS ver. 17.7).

**Figure 1.**
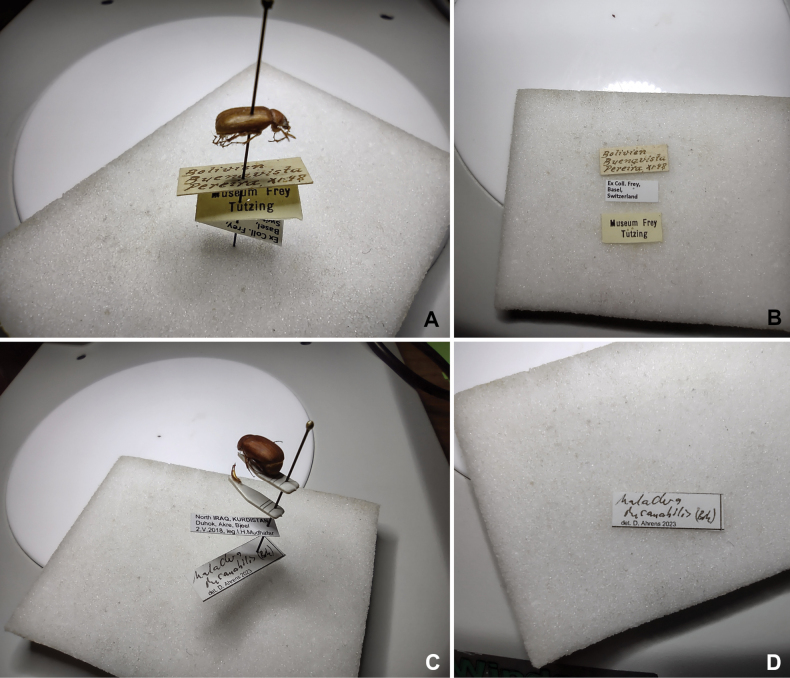
Exemplary specimens used for experimental real-time label scans **A** printed labels scanned on pin **B** printed labels scanned separately **C** partly handwritten labels scanned on pin **D** partly handwritten labels scanned separately.

We explored data extraction from labels with different approaches and alternative label conditions (Fig. 1). Some of the tutorials for each of these approaches can be found to be more suitable for different technical situations of the user than others. We describe subsequentially each of these approaches in simplified step by step tutorials. Tutorials are accompanied by screen shots and examples of retrieved data.

#### ﻿With any computer operational system and an Android mobile device:

##### Variant 1

1) Open the "*Google Translate*" or "*Google Lens*" apps in your mobile phone.

2) Focus on the label to be scanned and zoom virtually by using the touch screen so that the label fills the screen as much as possible. There is no need to be perfectly focused, but all letters should be recognizable.

3) Scan by clicking on the circle with the magnifier lens on it.

4) Mark the label text (Fig. 1) via cursor selection using the touch screen of the mobile phone.

5) Select “Copy to Computer”.

6) Confirm the selected device (computer with which you are logged into your Google account and via Google Chrome Browser) by choosing “Select”.^1^

7) On the computer: simply paste from clipboard into your target document (verbatim label citation).

8) Finally, you may proofread the scan (while having still your specimen in front of you) and manually correct misspellings or readings.

9) Finished.

Alternatively, in step 5 you may choose “Copy” and then paste this copied content into an open Google Docs document on the mobile device. This document can be accessed on via the same Google account on the synchronized computer. This step is sometimes necessary if the internet connection is too slow (see results below). This also works outside of the Google Cloud environment but is a little more complex: Files can be shared between Android, Windows or Mac devices using the "*KDE Connect*" app (https://kdeconnect.kde.org). The latter app works also on Linux. All devices must be in the same WIFI network. After installing the "*KDE Connect*" app, the text can be transferred to the computer.

##### Variant 2 (bulk scans)

1) Open the "*Google Keep – Notes and Lists*" app on your mobile phone.

2) Click on “+” and then on “image” icon.

3) Click on “Take photo” to capture the image, focus on the label you want to scan. Click on “photo” button, and then on the “checkmark” icon to save it.

4) Click on the image, then on the three dots in the upper right corner, and then on “grab image text”. The text will appear as a note and can be manually corrected for spellings or readings errors. A title for the note can be added. This function seems to work only on newer Android systems; here we used successfully Android ver. 13 and ver. 14. With an older Android ver. 10 or 11 smartphone, this option did not work.

5) Repeat steps 2–4 for each label you want to scan. They will be saved as separate notes.

6) Select all notes, click on the three dots in the upper right corner, and then on “copy to Google Docs” (This step can be alternatively done already on the computer via the respective google account; see Fig. 5). A single Google Docs document containing all images and texts will be generated. This step can be done on your mobile or on a computer logged into your Google account.

7) On your computer: open your Google Docs file, and the final corrections can be made and downloaded.

#### ﻿With an “Apple-only” environment

Requirements: Make sure you have a recent iPhone or iPad model with macro photography capabilities and the most recent operating system (iOS 15 and later). You will also need a Mac computer and an Apple iCloud account (at least the free version). An internet connection of the phone (e.g., via WLAN) is not necessary for data collection, if you collect your data from the specimen labels first on your phone (bulk scans) and go back to your Mac computer later.

##### a) Using "*Notes*" app:

1) Open the "*Notes*" app on your iPhone and set up a new note for your current project.

2) In your note, tap the camera symbol at the bottom and choose “scan text” from the pop-up menu. A camera window opens in the bottom part of your note.

3) Aim your camera at the text block you want to scan. Yellow brackets will show you which text block the software sees as target. Once the desired target text is within the brackets press the insert button at the bottom of the camera window. The targeted text will be read and automatically transferred to your note.

4) Briefly check the result in your note.

5) Go to the next line in your note and scan the next target text in the same way, thus accumulating information from multiple specimen labels or multiple specimens as you like.

6) Once finished with the data collecting, return to your desktop Mac computer. If the phone had telephone connection with your provider while you took the scans or on your way back to your desktop computer, the "*Notes*" app should automatically synchronize with your Apple Account in the background so that when you open the "*Notes*" app on your desktop computer, you should find all the scanned data there.

7) Continue to copy and paste the information accumulated in your "*Notes*" app to the document or database of your choice.

##### b) Using the Shortcuts app:

The "*Shortcuts*" app of iOS can be used to program an automated process from taking the photo, extracting the text and filling a table in Apple’s spreadsheet app "*Numbers*". Make sure that your "*Shortcuts*" and "*Numbers*" apps are synchronized for all of your devices via your iCloud drive. We assembled a "*Shortcuts*" algorithm as a proof of concept. Fig. 4 shows the algorithm.

#### ﻿Without internet connection, using Bluetooth with a Windows PC and an Android 14 mobile phone

1) Download the app ("*Google keeps – Notes and Lists*") on the mobile phone

2) Open Bluetooth options in the computer

3) Pair the computer and mobile phone

4) Click on receive files via Bluetooth

5) Open the app and click on the picture icon

6) Click on “take photo” and take the photo

7) Click on the captured picture

8) Click on the three dots in the upper right corner

9) Click on “grab image text” and select the extracted text^2^

10) Click on the three dots in the lower right corner and click on “send”

11) Click on “send via other apps” and choose the Bluetooth symbol

12) Choose a folder to save the html file in the computer

13) Copy the text from the html-file into a text editor for final spelling corrections

We expect this approach to work in a similar way also in Linux and Apple environments.

## ﻿Results

**Figure 2.**
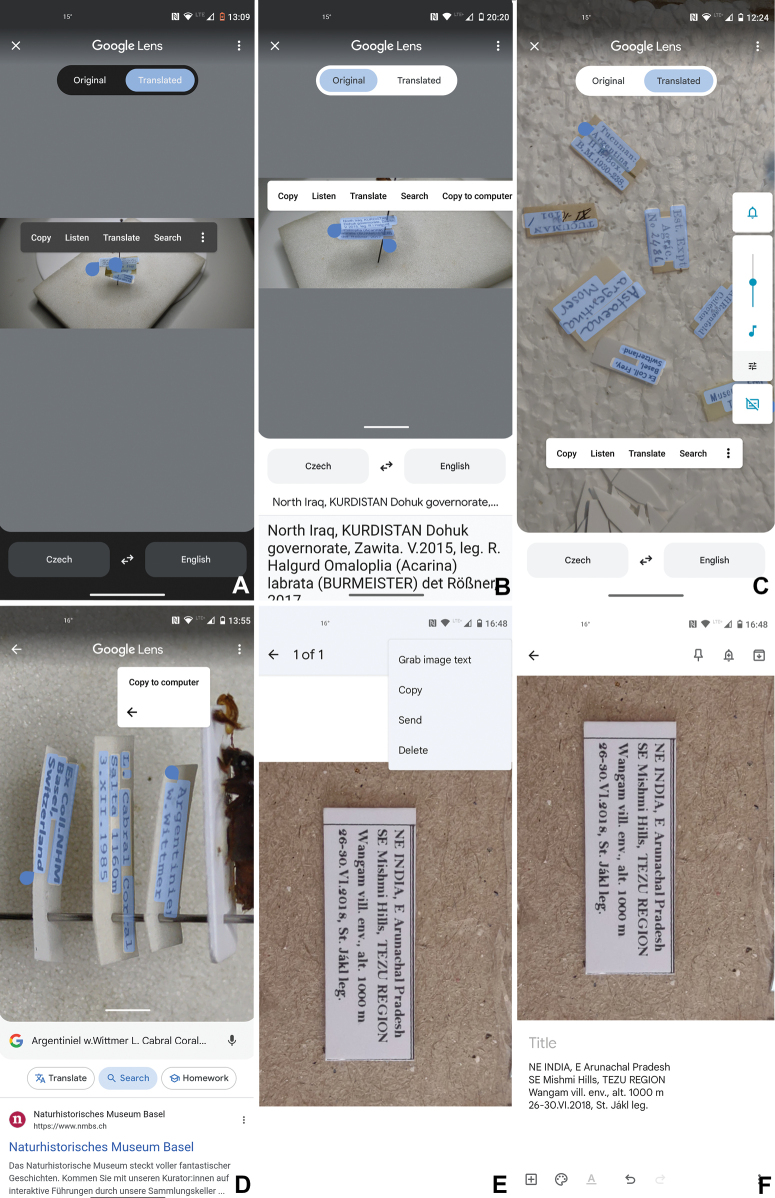
Steps of scanning (exemplified by a screenshot from mobile phone) of real-time data collection, and examples of labels **A** step 1: marking of the text to be captured via touch screen of the mobile phone (example - printed labels scanned on pin) **B** step 2: select from menu bar (at the right side under three dots) “Copy to computer” (example - printed labels scanned separately). As to be seen, different labels at different levels on the pin can be scanned simultaneously and do not need to be removed from the pin **C** Screenshot showing the capture of multidirectional printed labels scanned separately from the specimen in "*Google Lens*" **D** Screenshot showing the capture of multiple distorted, printed labels scanned on the pinned specimen in "*Google Lens*" **E** Screenshot showing the initial capture of a printed label scanned separately from the specimen in "*Google Keep*" **F** Screenshot showing the extracted data resulting from **E**.

In Table 1 we summarize the major characteristics of data capture using these methods. We show directly pasted content and the amount of real-time spelling corrections needed. In type-printed labels the corrections were minimal, but handwritten labels needed more correcting, depending on the size and style of the handwriting. In these cases, scanning the labels separately from the pin without distortion helped a lot (Fig. 1A, B). In printed labels, direction (Fig. 2C) and distortion of labels did not matter much (Fig. 2D). We were able to scan up to three labels (from the distorted side view) still mounted on a pin and without rotating the labels on, or removing them from, the pin (Fig. 2D).

**Table 1.** Summary of label configuration/ view (with reference to Figs 1, 2) and the obtained resulting text in the final database. Text corrected by real-time manual corrections are indicated in bold.

Label configuration/ view	Text as pasted from computer’s clipboard	Verbatim finalized data (after manual correction)
Fig. 1A (labels scanned on pin, distorted)	Belivr vista Peretra、インタ	“B**olivia Buenavista P**ere**i**ra **XI.48** / Museum Frey Tutzing/ Ex Coll. Frey, Basel, Switzer***land***” (CF).
	Museum Frey	
	Tutzing	
	Ex Coll. Frey, Basel, Switzer	
Fig. 1B (labels scanned separately, not distorted)	Bolivia Buengvista Pereira X198	“Bolivia Buen**a**vista Pereira X**I.4**8 / Ex Coll. Frey, Basel, Switzerland/ Museum Frey Tutzing” (CF).
	Ex Coll. Frey, Basel,	
	Switzerland	
	Museum Frey Tutzing	
Fig. 1C (partly handwritten labels scanned on pin, distorted)	North IRAQ, KURDISTAN Duhok, Akre, Bjeel 2.V.2018,	“North IRAQ, KURDISTAN Duhok, Akre, Bjeel 2.V.2018, leg.1.H.Mudhafar/ Maladera **insanbilis (Brsk.)** de**t**. D. Ahrens 2023”
	leg.1.H.Mudhafar	
	Maladera	
	del. D. Ahrens 2023	
Fig. 1D (partly handwritten labels scanned separately, not distorted)	Maladus dusanabilis (Boy)	Malad**era in**sanabilis (B**rsk**) det. D. Ahrens 2023
	det. D. Ahrens 2023	
Fig. 2C	Tucuman:	“Tucuman: Argentina. H.E.Box. Β.Μ.1930-238./ Est. Expt. Agric. No 2486/ TUCUMAN **XI-I** 1**9**1/ A H Rosenfeld Collector/ Astaena argentina Moser/ Ex Coll. Frey, Basel, Switzerland/ Museum Frey Tutzing“
	Argentina. H.E.Box. Β.Μ.1930-238.	
	Est. Expt	
	Agric. No 2486	
	TUCUMAN 101/	
	AHRosenfeld Collector	
	Astaena argentina Moser	
	Ex Coll. Frey, Basel, Switzerland	
	Museum Frey Tutzing	
Fig. 2D	Argentiniel w.Wittmer	“Argentinie**n W.** Wittmer/ L. Cabral Coral Salta 1160 m 3.XII.1985/ Ex Coll. NHM Basel, Switzerland” (NHMB)
	L. Cabral Coral	
	Salta 1160 m	
	3.XII.1985	
	Ex Coll.NHM	
	Basel, Switzerland	
Fig. 3A	四川:峨嵋山چہ	“四川:峨嵋山 1957.**VII.**31 中國科學院”
	19573131	
	中國科學院	

Low image resolution was not a problem, and we could zoom-in digitally, so the labels almost filled the screen of the phone. However, during our initial testing, we found that much smaller images were also successful in capturing data (Fig. 1A–D).

Processing time per specimen was fast, and we estimated that full data capture, including spelling corrections, was 3–10 seconds per specimen. Processing time was often a little longer for badly handwritten labels, when an insect pin or other labels covered parts of the label text, or when the internet connection was slow. The total time gain per label was larger with labels containing much information or with multiple labels. For example, in the labels shown in Fig. 2B, typing the data by hand into the computer required 60 seconds, including spell check, but scanning the label using Approach 1 took 8 seconds (including a manual spell check). For the data of Fig. 2C, manual typing and spell-check required 121 seconds, while Approach 1 took 10 seconds (including manual spell check). We refrained from more extensive comparisons in the time needed between manual typing and data scanning, because the former depends much on the typing skills of the person doing the data entry. The comparative numbers from a few test replicates given here, refer to a scientist without proficiency in typing (performed by D.A.).

In some instances, in Approach 1, we had to use the deviation via a Google Docs document due to bad internet connection, when the copy process failed due to slow data transfer. This was then usually two “clicks” (or seconds) slower, but not a major delay compared to the amount of time required for manual typing.

**Figure 3.**
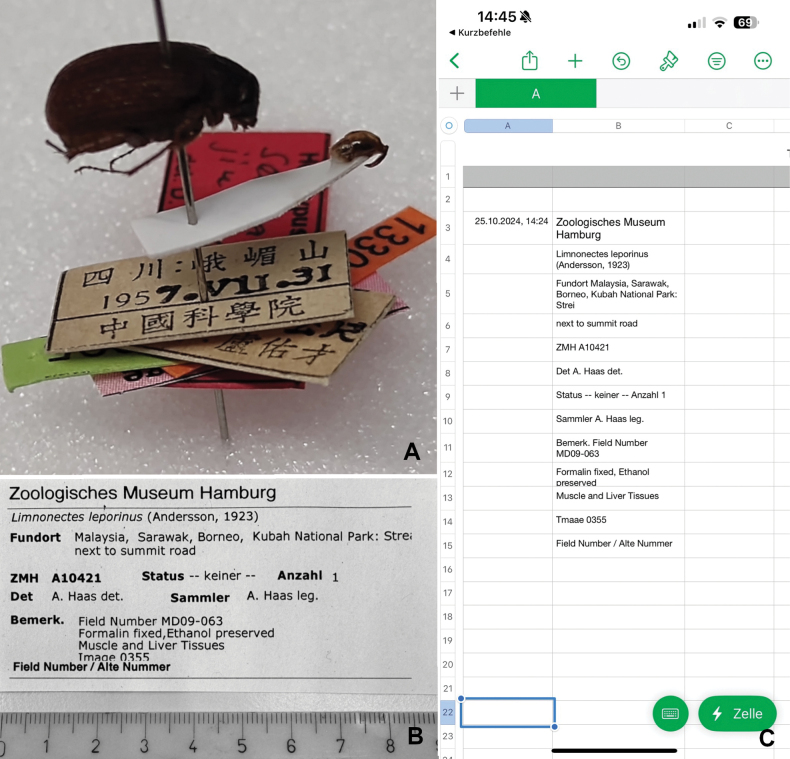
Other exemplary specimens used for experimental label scans **A** for Chinese language labels (printed) **B** printed Herpetology collection label that was scanned in the test of the Apple "*Shortcuts*" app algorithm. Note the incomplete text in the third text line and the cut off text “image 0355” below (compare to the corresponding data entries in C) **C** Screenshot of the automatically scanned collection label as transferred into cells of the spreadsheet app "*Numbers*". Although the text scan was very reliable, incomplete text will need editing: the somewhat cut off text “image 0355” of the label was interpreted as “Tmaee 0355”. The time stamp in the first column corresponds to the file name of the respective photo saved as backup in the Shortcuts directory.

**Figure 4.**
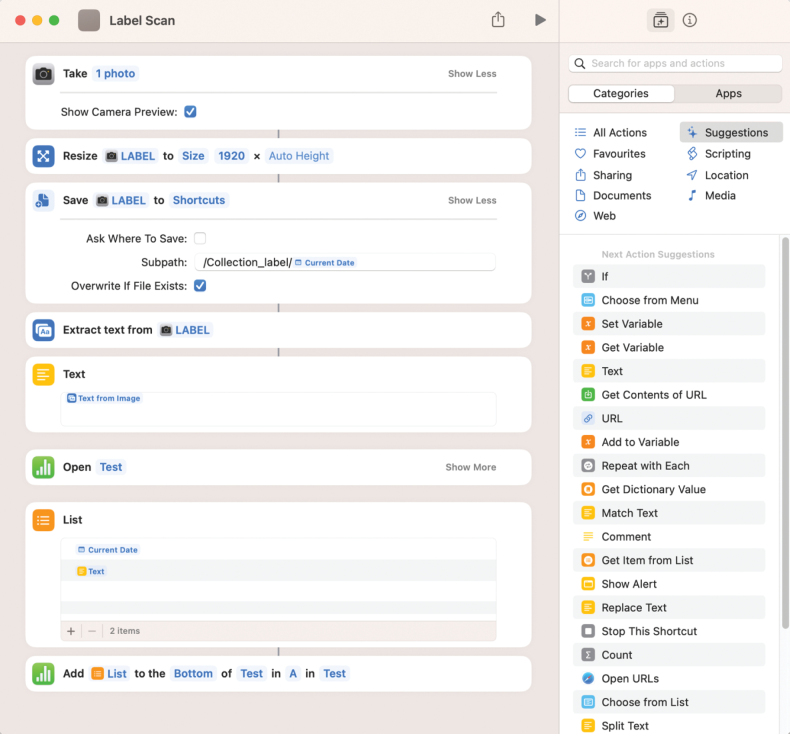
iOS "*Shortcuts*" app algorithm. From top to bottom: The first step will open the iPhone’s Camera app and lets you photograph the label. The photo (“LABEL”) is then resized (optional, to reduce space) and saved in the background to the "*Shortcuts*" directory in your iCloud account with the current date (and time) as file name. Then the text is extracted from the photo and stored to a text container. The next step opens the spreadsheet “Test” in app "*Numbers*"; an empty target spreadsheet file (here: “Test”) must be prepared beforehand and waiting in the "*Shortcuts*" folder of your iCloud account. Current Date and Text items are then collected in the “List”. The List items are finally entered into different columns in the spreadsheet file “Test” and a sheet with the name “A”.

The iPhone workflow test with was done with a larger label (Fig. 3B). In the workflow 2a) above, using the Notes app on the Apple iPhone, the image recognition tried to identify and focus blocks of text within the label, but did not to capture the label as a whole. To capture multiple bits of information the process had to be reiterated accordingly. Once the data has been collected in Notes, further copy-paste editing is necessary to transfer the data to a database. Workflow 2b), using "*Shortcuts*" app automation (Fig. 4) scans the whole label and also stores the data with a timestamp directly into a spreadsheet app. Furthermore, the photos are stored in the user’s Apple iCloud account (as backups for potential later reference), but this step is optional in the algorithm. The result of the scanning is shown in Fig. 3B, C. Note that incomplete text in the original caused interpretation problems (truncated third line and partially hidden bottom part of “image 0355”). In addition, the algorithm tends to place each recognized line of text in a separate cell. If several lines belong to the same block of information, editing of the cells was necessary. The scan of the label and filling of the cells in the spreadsheet took less than 10 seconds. The algorithm analyzed the label as lines of text and allocated one cell per line in the spreadsheet. This means that the locality information in our example was split up into two cells in our test. Depending on which further tasks the user wants to accomplish copy-paste processing of such splits is necessary.

The approach using a Bluetooth connection between the mobile phone and the computer appeared to be slightly longer (by the increased number of “device clicks”) than the direct approach (with the internet connection). Yet it saved a substantial amount of time for extracting the label data compared with manually typing. The use of Bluetooth may be necessary in situations where a good internet connection is unavailable, such as in collections. However, unfortunately the “grab image text” function of "*Google Keeps*" did work only with a newer smartphone (Android 13 or 14), not on an older device with Android 10 or 11.

**Figure 5.**
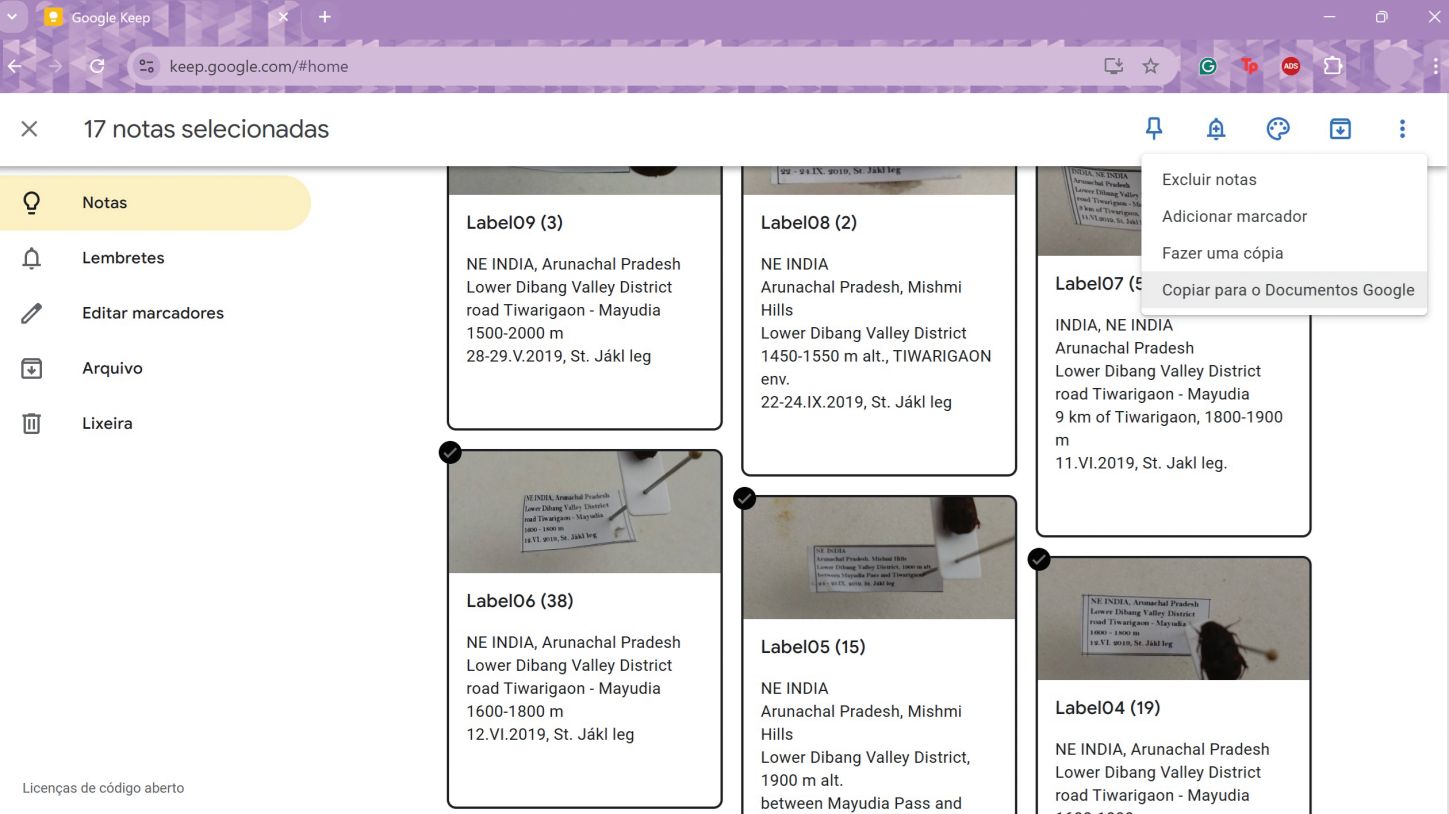
Screenshot of bulk-scanned labels via "*Google Keep*", inspected afterwards directly from the computer interface, during the step of copying to of the label text to a Google document (interface here in Portuguese).

Bulk approaches are available under the Google and Apple environment (Fig. 4) with the "*Google Keeps*" and "*Notes*" applications, respectively. In both, images are temporarily stored in the mobile devices, which can be later saved or discarded. While bulk approaches save time with data transfer, they have the disadvantage that potentially incomplete scans are only discovered when the specimens no longer at hand.

## ﻿Discussion

While new technologies, including artificial intelligence, are entering in our daily life, their use and application in biodiversity research is yet rather limited, although there have been developments in AI-powered label recognition (Johaadien and Torma 2023; Weaver et al. 2023; Takano et al. 2024). Herein, we addressed the scanning of data from labels using a smartphone using different operating systems. To our knowledge, this has not previously been explored and applied to insect collection specimens in particular. There are solutions for large-scale mass digitization of collections (Blagoderov et al. 2012; Tegelberg et al. 2014; Engledow et al. 2018; Belot et al. 2023). All these solutions require manual separation of specimens and labels to separately photograph them. Initial trials with robotic technology (e.g. Dupont and Price 2019) are promising, but such methods can only be used by larger institutions with the budget to do so.

With partly omitting the so far obligatory step of taking and permanently storing images of the labels, our direct approach to data capture is more rapid and environmentally more sustainable. In some of our procedures, data extraction happens without delay in the background, and there still is the option to retain the images if wanted. For a simple extraction of distributional data for taxonomic revisions or faunistic studies, we see no scientific necessity for long-term storage of images of specimen labels. Moreover, spell checking of the scanned and extracted data can be done when the specimen is at hand, and the data is finalized almost immediately.

However, depending on the individual needs and working conditions, the user has the choice on the individual workflow. It is possible to scan 50 labels in a row (i.e., bulk workflow) before transferring the data to the computer. Then in some critical cases, having a backup photo is good for quality assessment and spell checking.

Another great advantage is that these protocols use commercial devices that are simple to handle and which cost little to replace when they come into (informatic) age which is also a matter of cybersecurity. Unfortunately, in biosystematics, specialized devices are often overpriced, technologically obsolete, or require often expensive updates and service. Since biodiversity research in invertebrates, and especially entomology, is done in part by amateur scientists (and even professionals may lack funds for their “descriptive research”), funding may be lacking or limited.

Our results revealed that some functions may not be available on older smartphones running earlier operating systems. Here, the “grab image text” function did not work on Android 11 or older. Similarly, the other approaches might not work with even older devices, even if they work with the same app. However, we could not explore these limitations in detail, because we had only a limited selection of smartphones at hand.

The increasingly high reliability of text recognition and the rapid data transfer may make the use of machine readable barcode labels and QR codes superfluous in collection management, since connected data can be easily inferred from numerical voucher numbers on labels. Considering that optical character recognition (OCR) software, even when coupled with very advanced AI-technologies, possibly might give more errors than reading machine-readable codes (barcodes, 2D-codes), more rigorous tests are needed to check and compare the accuracy of a smartphone-based workflow compared to standard barcode and QR readers in this field.

Our solutions and tutorial proposed here are well suited for fast, secure recording of collection objects, e.g. when visiting a collection or when selecting individual objects. We are aware that habits, skills, and specific workflows influence the way we integrate such devices and text recognition capabilities. We are convinced that they will make a significant contribution and help to alleviate the taxonomic impediment (e.g., de Carvalho et al. 2005, 2007; Engel et al. 2021), as the workload for taxonomists capturing data of the material they study will be reduced by at least tenfold.

Finally, we note that there might be even more options and possibilities to scan labels with mobile devices. These options might evolve as quickly as mobile phones and artificial intelligence technology improve. Nevertheless, we expect that our paper will be an inspiration to others to continue exploring options on how to successfully apply this technology in their workflows and to share what they have learnt.

## Footnotes

This function works exclusively with the Google Chrome web browser.

This function works only on newer Android systems; here we used successfully Android v. 13 and v. 14. With an older Android v. 10 or 11 smartphone, this option unfortunately did not work.

## ﻿Additional information

### Author ORCIDs

Dirk Ahrens https://orcid.org/0000-0003-3524-7153

Alexander Haas https://orcid.org/0000-0002-3961-518X

Thaynara L. Pacheco https://orcid.org/0000-0001-9503-7751

Peter Grobe https://orcid.org/0000-0003-4991-5781

### Data availability

All of the data that support the findings of this study are available in the main text.
